# On the Nature and Treatment of Tic Douloureux, Sciatica, and Other Neuralgic Disorders

**Published:** 1844-07-01

**Authors:** 


### On the Nature and Treatment of Tic Douloureux, Sciatica, and other Neuralgic Disorders. By *Henry Hunt*, M.D. pp. 190. London, 1844.

There was a period when books were few in number, and he who gave one to the world might justly be termed a benefactor to his species. Their multiplication has now become a serious evil, and few are the authors entitled to national gratitude. This superabundance has generated the office of the Reviewer, an office we hold, although humbly be it spoken, if executed with a due sense of its responsibilities, of considerable importance to both authors and readers, indicating much that is valuable in the productions of the former, which else might never have been perceived by the latter, by reason of the physical impossibility of perusing all that has been written upon the subject, and the want of that discriminating tact which long practice can alone confer. An author entering the Court of Criticism must be looked upon, we do not say as a delinquent, but certainly as on his trial, charged with having written a book, and thereby of having increased the number already too considerable. It is for him to prove that such book supplies a want hitherto unprovided for, enounces a fact unnoticed, or recals attention to matters from which it should never have been diverted; and the exculpatory character of most prefaces proves that their writers are aware of this being their position. Medical readers have an especial claim that the precise object the writer has in view, and the degree of its attainment shall be inquired into, seeing that, compared to other classes, their means of purchasing, and time for perusing books, are both limited, while the temptation to acquire reputation by ministering to the ignorance and credulity of the public are very great.

These observations, however, are not very appropriate upon the present occasion, as Dr. Hunt assigns good reasons for publishing, namely, the having had numerous opportunities of witnessing the diseases upon which he writes, and his desire to place their treatment upon a less empirical basis.

"Having practised for many years in the South of Devon, the climate of which is proverbial for being warm, humid, and relaxing, and peculiarly calculated to produce the kind of habit most favorable for the development of these nervous diseases, which depend on weakness and relaxation, I had frequent opportunities of seeing and studying those diseases.

"My attention was early directed to tic douloureux, and after witnessing many cases, carefully tracing their origin, and comparing the various symptoms by which they were accompanied, I was led to conclude that this peculiarly painful malady had its rise in different individuals from different causes, and in very opposite conditions of the system; a conclusion that satisfactorily explained why a remedy, which had been successful in some cases, had completely failed in others.

"This has induced me to select several cases, in which I have been able to trace the pain to its source. These I have arranged under several heads, according to their respective causes: to each I have appended a few observations, and the means I have found most efficacious in their treatment."

He justly criticizes a very different plan, which is usually adopted by writers upon this subject, that is, the blazoning forth of some particular remedy as if it were a specific for the malady, no matter whence it arise, and its administration in such heroic doses as frequently to prove the means of aggravating rather than alleviating the original disease. Some of the most powerful articles of the materia medica have in this way been tried and found wanting, and that often from the injudicious mode of proceeding of the exhibitor, rather than from their own want of efficacy in properly selected cases. Many a medicine has been robbed of its just pretension to utility, and has sank into unmerited oblivion, to be revived sometimes under better auspices.

But the most scientific physician cannot always dispense with empirical remedies. It is true he is bent upon the investigation and removal of the cause of the disease; but, how frequently, when his researches are in vain, or his means of cure are but of gradual operation, do urgent symptoms (and what symptom can equal in overwhelming urgency the pain of tic douloureux?) oblige him to have recourse to this or that remedy which experience or accident have proved to have been sometimes useful in meeting them. Nay, more, his discovery of the cause of the disease would not *à priori*, lead him, on some occasions, to allow that the considerable extent of relief achieved by the remedy were possible; as is seen in some cases of neuralgiæ arising from organic and persistent causes, which are yet frequently much under the control of remedies that a knowledge of those causes would hardly lead us to anticipate benefit being derived from.

However this may be, there can be no doubt that Dr. Hunt has adopted the proper course for illustrating these affections in a satisfactory manner, and pointing out the extent to which our remedial power extends, and the best means of directing it. Mr. Swan, in his Treatise upon the Diseases of the Nerves, strongly insists upon a more discriminatory mode of treatment being adopted than is usually put in force; but we are not aware of any author who has presented so simple and useful a classification of the varieties of the disease as that contained in the work before us.

Omitting the description of the characteristic symptoms of the disease, we will pass in brief review the various causes capable of producing it.

1. *Tic Douloureux from some peculiarity of Constitution or Neuralgic Habit.*—Although many cases of the disease are frequently referred to this which a more diligent investigation of would discover to be attributable to more definite causes, yet there can be no doubt that there is, as the author states, " a class of persons, particularly females, in whom the natural sensibility of the nervous system is so increased as to render them peculiarly liable to neuralgic pains." In these sensitive beings, moral and physical impressions, which produce no ill effects upon ordinary mortals, induce, especially when their naturally feeble powers are lowered by any cause, some of the varieties of neuralgia.

In persons so predisposed, fatigue, anxiety, humidity of climate, the presence of malaria, &c. &c. easily induce the disease, and its removal is not to be accomplished unless these evil influences can be averted. To meet the debility by the direct application of tonics is frequently impossible, as in many cases these induce fever rather than relieve weakness. So too the subdual of the pain by the use of powerful narcotics is often bad practice, tending to obscure the discovery and prolong the operation of the original cause of its production. When, by the removal of various unfavourable circumstances, the patient is placed in a condition more propitious for her cure, time, patience, and perseverance are still required, and the danger of relapse must be borne in mind long after the period when precaution may seem to the unexperienced necessary. By attention to the digestive organs, the adoption of a judicious regimen, and the cautious use of mild tonics and sedatives, much may often be accomplished for fortifying the constitutional powers. Dr. Hunt places great reliance in the selection of a high, dry, open, but not bleak situation as a place of residence; and the pure air of the Welsh mountains has so invigorating an effect, that the patient requires cautioning not to imagine her powers greater than they really are, and by over-exertion induce fatigue, which is almost certain to bring on an attack of the disease.

2. *Tic Douloureux arising from Dyspepsia.*—The influence of a disordered condition of the nervous system in the production of dyspepsia is well known, and, on the other hand, the faulty performance of the digestive functions frequently proves the immediate cause of some of the most serious neuralgic affections. *Atonic* dyspepsia, as would be expected, is the usual form which prevails, and the *stomach* sometimes the only organ affected. Errors of diet, arising from too full or too sparing a regimen, may indeed alone call for remedying. Where the stomach is the sole organ in fault, Dr. Hunt has generally found the administration of an *emetic* the best preliminary, and has known many medicines prove successful after its employment which had long before been tried unavailingly. After clearing the stomach by this means and the bowels by a warm aperient, he gives *arsenic* combined with a sedative, " commencing with small doses, on the average of four minims, and gradually increasing the dose daily, until some decided symptom of its action is perceptible, which is commonly evinced, when the dose has amounted to 10." It is then to be suspended and afterwards recommenced in small doses, and requires to be continued for several weeks after the pain has disappeared, the neglect of which point by patients causes frequent relapses. As the treatment progresses, a grain of quinine twice or thrice daily will prove useful, although it would not have done so at the commencement. This plan of treatment Dr. Hunt has found far more successful than the administration of large doses of belladonna, iron, or quinine, and indeed it has proved so after these have been tried in vain, as they had been in many cases which came under his care. There are some cases, however, in which these small doses of arsenic seem to augment rather than allay the morbid irritability of the stomach; and in these the peculiar effects of the medicine are shown immediately, and not after gradually augmented doses, as in cases where it agrees. In such, it must be at once abandoned, and, as there is usually some inflammatory action present, a rigid diet, mild diaphoretics and small doses of prussic acid, aperients, and counter-irritation to the epigastrium be employed. After the morbid irritation is subdued, the arsenic may be again tried with caution, and often then succeeds, but if it still disagree it must be left off again and the case chiefly managed by a due attention to diet. Indeed, a regulation of *diet* in all these cases is of the first importance, for without its careful supervision and due adaptation to each case medicines will frequently prove useless.

But the dyspepsia is usually of a more complicated character, *the liver and other viscera* being in a condition of congestion: and in these more obstinate cases, the neuralgia, although an effect of the disorder of digestion at first, re-acts upon and increases the obstinacy of the primary affection. So too, many of the remedies employed to mitigate the pain, especially large doses of narcotics, derange in a great degree the various secretions. A primary object of attention is the relief of the infarcted state of the various abdominal viscera, and for this purpose no means can better first be used than a brisk *emetic*, which not only effectually empties the upper portion of the intestinal canal, but, by the action of the abdominal muscles and determination to the surface it necessitates, relieves much of the congestion of the various viscera, and moreover gives immediate though temporary relief to the agonizing pain, and exerts some effect in breaking the habit of its periodical occurrence. Mercurial purgatives must be repeated to the full unloading of the portal system, and a long space of time, or recourse to a mineral spa, may be required to effect this. In obstinate cases even mercurialization of the system, although generally to be avoided, may be required. It is in the class of cases we are now considering, that full doses of *croton oil*, as recommended by C. Bell, are most useful, acting as they do as emetics and brisk purgatives. Lately, small doses of the same drug with stomachic aperients have been found serviceable.

If the pain does not cease when a more healthy condition of the digestive organs has been procured, tonics or sedatives, properly adapted, must be employed. When the pain is intermitting and the system irritable, *arsenic* will be found useful; and when excessive pain and exhaustion are present, *belladonna* is the best sedative, especially when the pain occurs irregularly; while, when it is more periodical, opium and camphor may be given, about an hour before it is expected;—and the doses of these sedatives must be regulated by the extent of relief afforded, or the effect they produce on the system,—excessive pain causing a toleration sometimes of very large quantities.

Where the powers of the patient are very feeble, a more cautious and intermittent use of purgatives and an earlier use of tonics and sedatives may be required; and *arsenic* will be often found to be borne, when the various tonics are hurtful, although these will afterwards, when given in moderate doses, be found highly useful.

3. *Tic Doulourcux from Anæmia.*—The class of persons now to be considered, are contrasted with the first by the possession of a naturally strong constitution, but, having become anæmic, sometimes without obvious cause, become also afflicted with neuralgia. We must confess we think these cases are of rarer occurrence than the author supposes, and that originally low constitutional powers exist in the majority. The pallid and bloodless condition of these persons indicates the propriety of the use of tonics, and it is here that Dr. Hunt, without denying it possesses some efficacy in other varieties also, believes the *carbonate of iron* to be especially useful—requiring also sometimes the aid of belladonna, &c. to mitigate the severity of the paroxysm.

4. *Tic Doulourcux from Disease of the Spine.*—In this chapter the author draws the attention of the profession to a very important subject. After describing three interesting cases, he thus proceeds:—

"It was not until three or four similar cases fell under my notice, that I was led to conclude that the neuralgia of the cheek was only the effect or consequence of morbid action in the spine; but the history of the foregoing cases, and still more the effect which the remedies applied to the spine had on the neuralgia, were so striking, that it would be difficult to doubt their connection. * * *

"It is now universally allowed that for a considerable period, more especially from 10 to 20 years ago, numerous young persons were considered to be suffering from disease in the spine, were confined to their couches or beds, and tormented by cupping, leeches, issues, setons, &c. to the serious injury of their health; whilst the pain in the spine and the accompanying, rather than consequent, debility was the result of disorder of the general health, requiring the adoption and firm perseverance in a plan of treatment directly contrary. I think, however, that it must also be admitted, that of late years the opposite error has been fallen into, with far more serious results.

"It cannot be doubted that inflammation and real disease in the spine have, in numerous cases, been overlooked, and that the pain in the back has been considered nervous or hysterical; the incomplete palsy, to a want of volition in the patient; and that attention only to the general health, forced exertion, fresh air, &c. have been persevered in until the disease in the spine has attained to such a height, that the bones and cartilages, in some cases, but more commonly the spinal cord and its envelopes, have become disorganised, and protracted misery or death has been the result.

"Examples of this kind have fallen within my own practice, and several other instances have occurred in that of others, who have mentioned the cases to me.

"From anxious and close attention for many years to a considerable number of these cases I have become impressed with the conviction that many come rightly under both definitions. I mean, that in the commencement, and for a long period after, the pain in the spine is purely nervous or hysterical, depending upon some morbid condition of the body generally: but that, after an indefinite time, and when it has not yielded to the treatment adopted, the disorder degenerates gradually into real disease: the state of nervous excitement in the part inducing increased vascular action, a low kind of inflammation, which ultimately produces the disastrous consequences before mentioned." 79.

5. *Tic Douloureux from Disorder of the Uterus.*—Many of the cases comprehended by the author under the above head, would be more correctly included under that of anæmia, originating as they do in uterine hæmorrhage or profuse leucorrhœa—the ordinary sources of the anæmic condition. He alludes to another class of cases however, viz. the toothaches of pregnancy, which occasionally approach tic douloureux in severity, which can only arise in consequence of the morbid sympathies excited in the system by the gravid uterus. So, too, many young delicate women suffer severely from affections of the nerves of the face just prior to the menstrual periods. Of all remedies *arsenic* seems best adapted to neuralgia arising from excessive discharges, as not only allaying the morbid irritability of the nervous system, but also exciting a powerfully restraining effect upon the passive uterine flux: the various other usual local and general remedies, which we need not detail, being also attended to. Quinine, after the cessation of the discharges and relief of the pain, exerts a powerfully strengthening effect upon the constitution. Steel and other tonics, if given prematurely to relieve the mere debility, often do harm, by exciting febrile action and augmenting the menorrhagia.

6. *Tic Douloureux from Disease of the Brain.*—These cases are frequently not capable of correct diagnosis, in consequence of the length of time the pain may continue intermittent and the temporary success of remedies. The true state of the case is ultimately revealed by the persistence of the pain and its conjunction with other marks of cerebral disease.

7. *Tic Douloureux from Local Mechanical Causes.*—These cases are by no means of infrequent occurrence, although they are sometimes of difficult detection. Among them may be enumerated unnatural growths of bone, tumors along the track of the nerves, local injuries of nerves, foreign substances irritating them, diseased condition of the teeth, &c.

The *teeth* indeed should be always carefully examined, not only during a paroxysm, but when it is not present. During the paroxysm some of the teeth are always severely affected, even when the disease arises from other causes. If a particular tooth be that in which the pain always commences, if it is especially sensitive during the paroxysm, and pressure on it always excites a paroxysm, it should be removed. Teeth apparently sound have their fangs sometimes in an unnatural condition. Small portions of fang left in after extraction sometimes give rise to the disease and should be removed. The author adds :—

"It may not be irrelevant here to remark that, the teeth are more frequently the sufferers from tic douloureux, than the cause of it. I have often observed that the teeth of persons, long afflicted with this disease, however good and firm they may have been originally, become suddenly afflicted with caries, which does not appear to touch the crowns of the teeth, but merely that point at the edge of the gums, at which the enamel begins. Many teeth, indeed, the majority of those in the jaw, the seat of pain, have become affected almost simultaneously. Whether this is to be attributed to the deranged state of the general health, or to the repeated excitement in the teeth themselves, is uncertain. That this disease in the teeth does not produce the neuralgia is evident, from the fact that the teeth do not always become unsound until after the neuralgia has been of long-continuance, and sometimes not until it has been cured." 112.

8. *Tic Douloureux from Malaria and other Causes.*—Dr. Hunt, referring to Maculloch for the illustration of the disease as produced by malaria, briefly alludes to the fact of its sometimes following the *recession of cutaneous eruptions,* and irritation of the lower intestines.

*Periodical Headache.*—This is a common enough disease, especially in aguish localities and in persons suffering from debility. It may, however, attack the robust. It occurs sometimes during pregnancy, but more frequently after undue lactation and anxiety of mind. Those who have once suffered become exceedingly susceptible of future attacks from very slight exciting causes. The disease is generally easily curable if the case be recent. *Emetics* are usually indispensable, and other medicines frequently fail until they have been given. After the due operation of these and purgatives, quinine or arsenic should be given. Large doses of the former are said to cure quicker, but the author prefers more moderate ones as safer, although requiring to be continued longer. The *arsenic* should be given in small doses, and gradually increased until its effect on the stomach becomes apparent, when the pain almost always ceases, after which the medicine may be suspended for a day or two, and then renewed for a few days, or relapses will occur. A combination of capsicum or black pepper with the quinine or arsenic sometimes cures rapidly; and in some obstinate cases the arsenic in substance has succeeded where the solution has failed. Local anodyne applications are sometimes useful. A change of air, either during or immediately after the cure of the complaint, is of great service.

*Sciatica.*—Dr. Hunt justly insists upon the necessity of distinguishing carefully between the inflammatory and non-inflammatory forms of this disease, which however it is sometimes difficult to do, as it may be inflammatory in one stage, and simply neuralgic in another. The common cause of the acute inflammatory sciatica is the exposure of the lower part of the person to wet and cold. Free cupping and purging must be resorted to, the purgative being combined with colchicum, and followed by Dover's powder. Bleeding and purging will not however always relieve the pain, and must not be continued after the inflammatory action is subdued. Combinations of Dover's powder, calomel, and colchicum are to be continued, with additional opiate at night if required. Successive blisters may be necessary, especially behind the trochanter, and, if the affection yields slowly, one of them should be dressed with mercurial and savine ointment. The mercurial action may be required to the extent of slightly affecting the mouth.

The disease treated actively is usually prevented becoming *chronic;* but this condition may occur without having been preceded by an acute stage. As there is little febrile action, and the pain intermits, it is often difficult to distinguish this case from one of pure neuralgia. The occurrence of a kind of nocturnal fever, with an exacerbation of pain when warm in bed, sometimes assists in the diagnosis, as does the examination of the urine, which is usually scanty and lateritious in the inflammatory, and pale and abundant in the neuralgic form. In both, the limb frequently becomes powerless and wastes. The disease sometimes comes on very slowly in those exposed to wet and cold, the pain not being severe, except at night, and the limb only lame and weakly; but the general health gradually participates in the mischief. *Mercury* should always be resorted to, where circumstances do not urgently forbid it, and Dr. Hunt especially recommends the following formula:—℞. Hydr. phosphatis, gr. j., Opii gr. j., Ant. tart. gr. $\frac{1}{6}$, which he gives every night. But when the patient has been long ill before he is seen, and especially if he is advanced in years, the effectual clearing out of the lower bowels must be particularly attended to, not only as a proper preliminary for any treatment, but as operating a complete cure in some cases alone. While the mercury is taking, moderate doses of colchicum and nitrate of potass, mild aperients, sarsaparilla, change of air, &c. according to the requisites of each case, may be employed, and effectual counter-irritation by blister, tartar-emetic, or seton, put into force. The author has found iodide of potassium a useful remedy in the few cases in which he has as yet tried it.

The severe *pain* often requires special remedies, of which opium is usually the best, although Dr. Hunt has found a combination of belladonna, stramonium, and hyoscyamus, a very good one. These remedies are especially useful in the true *neuralgic* form of the disease. This may succeed chronic inflammation, or may come on unpreceded by it. The pain is agonizing, occurring at intervals, sometimes periodical ones, during the day, but not with nocturnal exacerbation. When the disease is not traceable to any particular cause, it should be treated in the mode already mentioned as applicable to the tic douloureux. This form is comparatively rare, and the paroxysm has been sometimes averted by large doses of oil of turpentine. It is in this form that acupuncturation proves unquestionably useful, providing there be no inflammation or heat of the parts when the needles are applied.

Sciatica may also be induced by the irritation of piles, prolapsus ani, or the pregnant uterus; and, although the affection usually subsides upon the relief of these conditions, at other times it remains a serious malady.

*Intermittent Neuralgia.*—Among other practical observations are the following:—

"The type usually assumed by neuralgia, is either the quotidian or the tertian, more rarely the quartan, or double tertian. Sometimes the paroxysms return three times a day: occasionally every second or third hour throughout the night, as well as the day: sometimes they recur at the expiration of eight days, a fortnight, or a month; and in some cases, annually, in particular months.

"In the periodical character which it assumes, neuralgia resembles ague: the type varies in the former as well as in the latter; a quartan changes into a tertian; a tertian into a quotidian; and sometimes an intermittent neuralgia becomes remittent.

"No explanation, I believe, can be given, why one form of intermittent should change to another, a tertian become a quotidian, and the reverse; but there is reason to believe, that, when an intermittent becomes remittent, it is to be attributed to some alteration in the condition of some of the viscera. This opinion is confirmed by the effect of remedies given for the cure of it. I have seen cases of tic douloureux, originally intermittent, which had become remittent, and again assumed the former type; in some, after an emetic and brisk purging; in others, after a free use of calomel, sufficient to affect the system." 155.

If, when the disease has become remittent, the medicines which had proved useful in its intermittent state be persisted in, especially bark and quinine, harm will result. Deobstruents and evacuants must be employed until the visceral engorgement be subdued, after which the anti-periodic may be usefully resumed, occasionally interposing a purgative.

*Sedatives.*—Employed usually on their introduction into practice as the principal means of cure, these are now looked upon only as valuable auxiliaries, not to be needlessly engaged, and to be dismissed on the earliest safe opportunity. Dr. Hunt states the extract of *belladonna* to be one of the most valuable, a full dose being a grain, which he has never repeated oftener than for three-hourly doses, two of these ordinarily giving relief. It is well known that, in these cases of excessive pain, full doses alone are of use, so that almost incipient poisonous effects have to be produced before relief is obtained.

"That large doses are necessary to produce these effects, and do not cause any of the dangerous symptoms sometimes to be anticipated from them, may be thus explained. The state of the system on which the pain depends, has either the power of resisting the action of these medicines, or a certain amount of their power is expended in bringing the nervous system to its normal state, and until that is effected, the usual symptoms of their action will not be developed; that, however, being effected, a small additional quantity will produce the symptoms above alluded to; which will clearly indicate that the required quantity has been administered, and that relief will soon be afforded, or must not be expected from them." 161.

The author prefers giving a full ordinary dose, very frequently, to administering a very large one at once, or gradually increasing a small one. Thus the sedative may be given every two, or even every one, hours, with far more safety than in one of the other modes, and with more certainty than in the other. When relief has been effectually obtained, the medicine should be suspended, and a smaller dose will in future suffice to keep the pain in check. A third or half a grain of belladonna may be continued two or three times a day, with a larger one at night, in proportion to the degree of pain and irritability which may remain. As the case improves, the medicine must be still further diminished, and the patient instructed so to take it as to anticipate the paroxysm, when possible.

Sedatives are most useful in the cases of genuine neuralgic habit, where irritability and debility predominate; and in such they must be continued, in small doses, even after the pain has been subdued, gradually diminishing these, and replacing them by invigorating measures, as the improved condition of the symptoms permit. They are very useful, given just before the menstrual periods, in neuralgia, arising from menorrhagia and other uterine disorders. When there is morbid sensibility of the intestinal mucous membrane, although sometimes useful, in small doses.

combined with ipecacuanha, or hydr. cum creta, yet, in most cases, they are hurtful. When the urine is pale, copious, and of low specific gravity, they may usually be given with advantage. In a congested condition of the abdominal viscera they do much harm, until, at least, these have been unloaded by purgation, &c.

*Sesqui-oxyde of Iron.*—This is a valuable medicine in the anæmic condition, and occasionally in other cases dependent upon no obvious cause. It would seem to cure by giving tone to the system, thus economising the predisposition arising from debility; and is, therefore, sometimes advisable, in cases where the pain has already been relieved by arsenic, sedatives, &c. in order to aid in rendering the cure more permanent.

The immensely large doses in which it has been given, are useless or prejudicial, and it usually requires to be continued for several weeks before benefit is derived from its employment.

*Arsenic.*—Dr. Hunt places great confidence in the beneficial effects of this medicine in properly selected cases, when it is judiciously administered.

"Arsenic operates most favourably on persons who are of lax fibre, accompanied by a languid state of the circulation, and whose secretions are rather profuse than otherwise; the urine pale and plentiful, and more especially on those whose skin is cold and moist. In persons of this description, whilst arsenic, to an extent far beyond other medicine, relieves the neuralgic pain, it improves the general health, and gives firmness and vigour to the constitution.

"I do not mean to assert, that arsenic invariably disagrees with persons of an opposite condition of the system; but when the urine is of a deep colour, and scantily secreted, or when it deposits the lithate of ammonia; the tongue loaded, especially if the tip or edges of it are red; arsenic almost invariably disagrees, and aggravates the pain. But as this morbid state of the system frequently depends on, or is complicated with, disorder of some important viscus, arsenic will often agree, and relieve the neuralgic pain, after the visceral disorder has been removed by appropriate remedies.

"Between these extreme states of the system, there are many shades of difference, which require modifications both in the general treatment, and in the use of arsenic: which it would be needless, were it possible, to describe." 174.

Arsenic is peculiarly indicated in neuralgia from malaria, the neuralgic habit, and in that form which arises from morbid sensibility of the stomach or uterus. It is usually hurtful when the cause of the pain arises from spinal diseases, anæmia, local disease, or injury of the nerves and an engorged condition of the abdominal viscera.

When the bowels have been thoroughly cleared out it may be given in doses of four minims of the liquor three times a day, and in some cases only in two minim doses. A minim to be added daily, until the peculiar effects of the medicine begin to shew themselves, when the dose should not be further augmented, and the medicine employed for a few days longer, and then suspended, to be again resumed, in the small doses, if the pain recurs. The disagreeable effects it produces on the skin, stomach, &c. must not be excited to a great extent, and the medicine can seldom be used for a long period without occasional suspension. Where the liquor has failed, the arsenic in substance, rubbed down with a little black pepper, and carefully divided, has, in doses of $\frac{1}{10}$ or $\frac{1}{20}$ of a grain, often succeeded in relieving the disease.

Dr. Hunt considers that the danger arising from an accumulation of arsenic in the system has been exaggerated. The condition of the urine often alone indicates the propriety of suspending it.

> "I have often observed, that one of the earliest indications of its operation is the change in the state of the urine. The urine is less plentifully secreted, becomes more acid, and of a deeper colour; and precipitates, if the medicine be continued for any length of time, the lithate of ammonia. Should no other indication be manifested after arsenic has been given for some time, and in pretty large doses, the use of it should always be suspended, when this alteration of the urine is perceived."

*Local Remedies.*—Upon these the author places little reliance, although employing them occasionally as means of relieving the pain. Their operation is uncertain, arising, in some measure, as he observes, from the different condition of the nerve, at the seat of pain, where they are applied, as regards tenderness, heat, &c. The *aconitine,* one grain to the ounce, produces a temporary palsy of the nerve, but the pain returns when this effect has subsided. *Belladonna,* either alone, or combined with ung. picis and creosote, or a poultice of linseed meal and bruised *stramonium* seeds, have been found also temporarily useful. *Veratria* the author has never found so, even when employed in the proportion of ten grains to the ounce.

In conclusion, Dr. Hunt, remarks, that he is well aware there are other causes of these neuralgic affections besides those he has alluded to, but which from their obscure nature too often defy successful investigation. In the mean time, we can only correct every apparent disorder of the system; and by diligently observing the various phenomena endeavour to obtain a clue to their origin and successful treatment. Our object should be not only to examine into the various exciting causes of the disease, which have been now alluded to, but to endeavour to discover the nature of the constitutional peculiarity upon which their influence depends; from among the vast number of persons who are subjected to them, only a small proportion, owing to the operation of such peculiarity, become afflicted with neuralgia. So, in treating the case, the ostensible relief of the affection is not sufficient; and we must, by amended habits of life, judicious diet, and persevering attention to a variety of apparently minute circumstances, strive to eradicate the delicacy of frame or other peculiarity in which the disease seems primarily to originate; and, in proportion as we succeed in our efforts, will the security against relapse be obtained.

The work, as we have already observed, is valuable, as containing a correct estimate of the amount of our present knowledge concerning this painful and interesting class of diseases, and indicating the mode by which this can be most effectually augmented. Each division of the subject is illustrated by some very interesting cases. We cordially recommend the book to our readers.

